# Optimizing sequestered carbon in forest offset programs: balancing accounting stringency and participation

**DOI:** 10.1186/s13021-019-0131-y

**Published:** 2019-12-03

**Authors:** Lindsey Wise, Eric Marland, Gregg Marland, Jason Hoyle, Tamara Kowalczyk, Tatyana Ruseva, Jeffrey Colby, Timothy Kinlaw

**Affiliations:** 1 Department of Mathematical Sciences, Appalachian State University, Boone, USA; 2 Department of Geological and Environmental Sciences, Appalachian State University, Boone, USA; 3 Appalachian Energy Center, Appalachian State University, Boone, USA; 4 Department of Accounting, Appalachian State University, Boone, USA; 5 Department of Government and Justice Studies, Appalachian State University, Boone, USA; 6 Department of Geography and Planning, Appalachian State University, Boone, USA

**Keywords:** Carbon accounting, Forest offset, Sequestration program

## Abstract

**Background:**

Although there is broad agreement that negative carbon emissions may be required in order to meet the global climate change targets specified in the Paris Agreement and that carbon sequestration in the terrestrial biosphere can be an important contributor, there are important accounting issues that often discourage forest carbon sequestration projects. The legislation establishing the California forest offset program, for example, requires that offsets be “real, additional, quantifiable, permanent, verifiable, and enforceable”. While these are all clearly desirable attributes, their implementation has been a great challenge in balancing complexity, expense, and risk. Most forest offset protocols carry similar accounting objectives, but often with different details, (e.g. Richards and Huebner in Carbon Manag 3(4):393–410, 2012 and Galik et al. in Mitig Adapt Strateg Glob Change 14:677–690, 2009). The result is that the complexity, expense, and risk of participation discourage participation and make it more difficult to achieve climate mitigation goals. We focus on the requirements for accounting and permanence to illustrate that current requirements disproportionately disadvantage small landowners.

**Results:**

The simplified 1040EZ filing system for U.S. income taxes may provide insight for a protocol model that balances reward, effort, and risk, while still achieving the overall objectives of standardized offset protocols. In this paper, we present initial ideas and lay the groundwork behind a “2050EZ” protocol for forest carbon sequestration as a complement to existing protocols.

**Conclusion:**

The Paris Agreement states that “Parties should take action to conserve and enhance, as appropriate, sinks and reservoirs of greenhouse gases.” The Paris Agreement also refers to issues such as equity, sustainable development, and other non-carbon benefits. The challenge is to provide incentives for maintaining and increasing the amount of carbon sequestered in the biosphere. Monitoring and verification of carbon storage need to be sufficient to demonstrate sequestration from the atmosphere while providing clear incentives and simple accounting approaches that encourage participation by diverse participants, including small land holders.

## Background

### The role of forest carbon sequestration

As we continue to struggle toward meeting a 2 °C (or less) limit for raising global average temperature [1, 2], there is wide agreement that the only avenue to success is to include negative CO_2_ emissions strategies [1, 3, 4]. Simply reducing emissions is likely not enough. One of the most effective ways to achieve negative emissions is by sequestering carbon in the terrestrial biosphere (including soils), and then increasing the time it takes for that carbon to make its way back to the atmosphere. This can be achieved in a variety of ways, from reforesting land currently without trees, to increasing the growth of existing forests and preserving forest land likely to be cleared, to increasing the mean lifetime of harvested wood products. While carbon sequestration represents negative emissions it is often presented in the mitigation literature as offering offsets while existing positive emissions are reduced.

While not without risks and uncertainties, reforesting, improving forest management, protecting forests, and increasing the life of harvested wood products increase the overall stock of carbon pulled out, and kept out, of the atmosphere.

If enough carbon is going to be stored in forests around the globe in time to keep warming levels to the 2° target, this needs to be initiated soon and on a large scale (e.g. [5]). At the same time, it takes time for a cultural shift to take place, and gaining the needed support from landholders where carbon can be sequestered is important. Incentives can be offered to increase participation by making it more appealing to grow or maintain forests. Understanding the motives and objectives of landowners can give us insight into constructing incentives that will work for a variety of different motivations, motivations that vary by country, by region, or even within regions by the size of landholdings and the demographic characteristics of landowners (e.g. [6, 7]). Land ownership varies from countries where essentially all forest land is publicly owned (e.g. Russia and Thailand) to countries where the dominant control over forest land is by private owners (e.g. 58% private in the U.S. and 75% private in Sweden in 2010) [8].

### Incentives and offsets

The Paris Agreement [2], now signed by 195 Parties and ratified by 183 (as of 5 April, 2019), cites the need to pursue actions for carbon sequestration and encourages incentives for sequestration activities [9, 10]. The Agreement does not specify a method or an accounting approach, leaving these open to discussion and further specification.

Current strategies include the ideas of carbon taxes, cap-and-trade systems, carbon offset markets, and small scale investing in urban forests, among other activities. There has even been the suggestion of; growing trees to bury in the ground where they cannot oxidize back to the atmosphere (e.g. [11]).

Working toward increasing negative CO_2_ emissions, some accounting issues present challenges to broad participation in current programs. While we focus this discussion on the details of the California [12] program, many programs for reducing carbon emissions attempt to supplement these reduction efforts with sequestration “offsets” (i.e. negative emissions), but then have to deal with the question of the numerical equivalence of emissions reductions and offsets. With reductions for uncertainty, leakage, reversion risk, and land value (avoided conversion), offsets earned are not the numerical equivalent of tons of carbon sequestered, making a comparison to emissions debatable. In an attempt to prevent loopholes and clarify procedures, offset programs can make participation subject to stringent requirements and detailed reporting that make offsets only economically viable to a limited set of landholders, or appealing only to the altruistic. Kerchner and Keeton [13] estimate that the California forest offset program, for example, is not financially viable for projects less than about 600 ha.

The legislation establishing the California forest offset program [12] requires that offsets be “real, additional, quantifiable, permanent, verifiable, and enforceable”. While these are all clearly desirable attributes, their implementation has been a great challenge in balancing complexity, expense, and commitment. Participation in this program is low relative to the number of potential participants [14]. Accounting also encounters issues of leakage since only broad participation in a closed system can insure against sequestration in one area being negated by a responsive emission elsewhere.

Carbon incentives for the land sector can be structured in different ways. (1) Practice-based payments provide funding to support a variety of conservation programs, or (2) Pay-for-performance programs wherein landowners are compensated on the basis of how much carbon they actually sequester, in some cases generating tradable carbon credits. The Paris Agreement states simply that “Parties should take action to conserve and enhance, as appropriate, sinks and reservoirs of greenhouse gases” [2, Article 5.1]. The Paris Agreement also moves beyond carbon and refers to issues such as equity, sustainable development, and other non-carbon benefits. We must consider how the 17 U.N. Sustainable Development Goals [2] factor into such efforts. Osborne and Shapiro [15], for example, describe the relative successes of two carbon sequestration projects in Mexico when one is tightly focused on carbon accounting and the other has less rigorous accounting but is cognizant of social and environmental co-benefits.

The challenge is thus to provide incentives for maintaining and increasing the amount of carbon sequestered in the biosphere while simultaneously pursuing the other social and environmental goals of the U. N. Sustainable Development Agenda and of program participants and program neighbors. Monitoring and verification of carbon storage need to be sufficient to demonstrate additional sequestration from the atmosphere. Motivations need to have adequate near-term focus to confront current environmental and market changes and to avoid foreclosing options for the future.

This short paper raises the question of motivating participation in forest offset programs that would involve increasing participation by adopting less stringent program requirements, or by reassigning responsibilities such as permanence and additionality from the project level to the programmatic level. We focus on “permanence” but speculate that less stringent standards generally would increase participation by small landowners or financially marginal parties. We suggest that overall program impact could be increased by balancing ease of participation with the rigor of accounting and verification and that rigorous program requirements disproportionally discourage small landowners.

## Methods and results

### The importance of small landowners

**Fig. 1:**
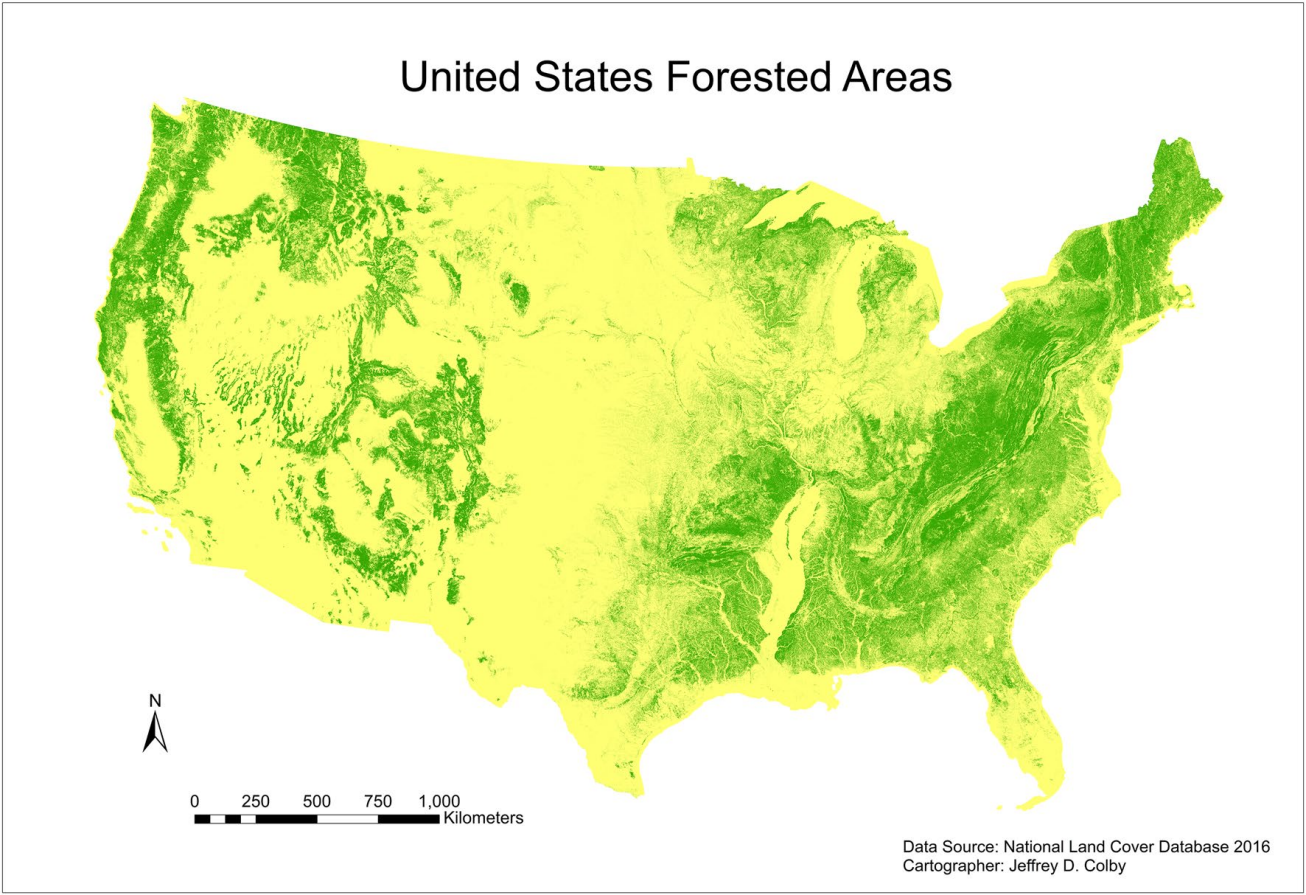
A map of forest land in the contiguous U.S. shows the broad suitability for forest cover and the broad potential for maintaining and/or increasing carbon sequestration in forests

**Fig. 2:**
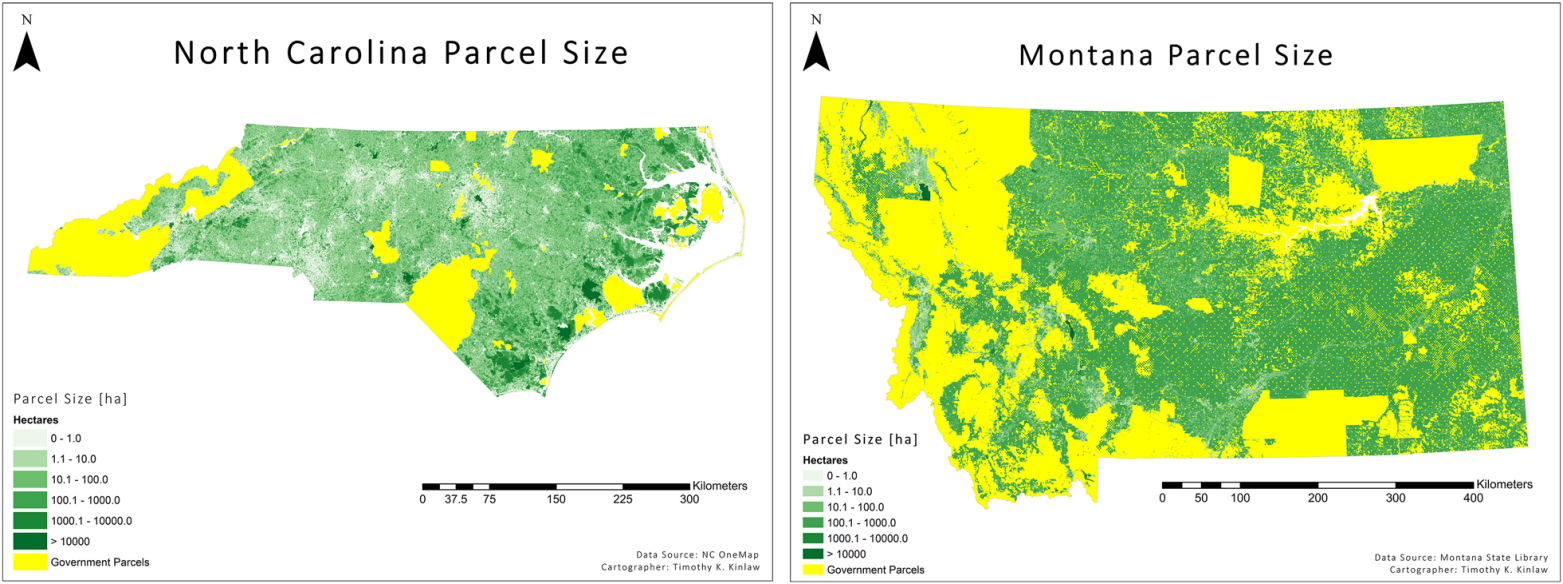
Maps of U.S. states North Carolina and Montana [17] illustrate land parcel size for the states. Yellow colored parcels are government owned. The rest of the parcels are shaded by parcel size with the darker green indicating larger parcel sizes. Data are from the NC ONEmap [18] resource produced by the North Carolina Centers for Geographic Information and Analysis [19]

Many barriers to entry with regard to the commonly cited requirements disproportionately affect small land owners (landowners with less than about 200 ha), who own more than 50% of the private, forested land in the United States. Data from 1994 show that more than 90% of private owners of forested land had holdings of less than 40 ha and that these smaller parcels involved over 30% of private, forested land in the U.S. [16]. Figure 1 shows the extent of current forest land in the contiguous U.S. and suggests the broad suitability for increasing forest coverage or improving forest management to increase carbon storage. Figure 2 shows the distribution of land ownership parcel size (including unforested land) in the U.S. states of North Carolina and Montana, illustrating the dominance of ownership by small landowners in North Carolina and of slightly larger parcel sizes in Montana.

**Fig. 3:**
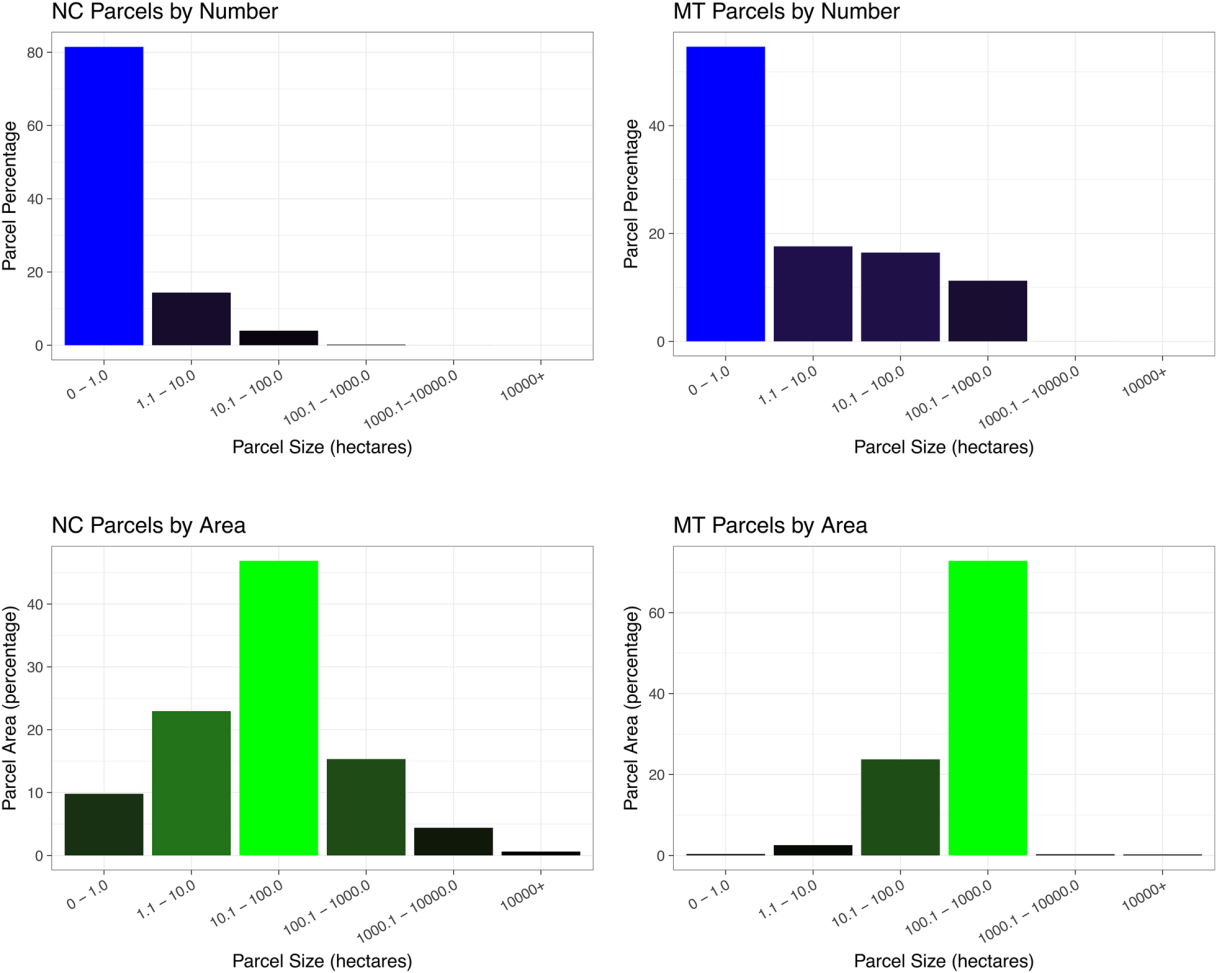
Histograms showing the distribution by parcel size of land ownership and land area in North Carolina. The top two panels reflect the proportion of owners with different parcel sizes while the bottom two panels reflect the proportion of total area in the state taken up by parcels of that size category. Data is from the NC ONEmap resource produced by the North Carolina Centers for Geographic Information and Analysis [19]

With the suggestion above that parcels below 600 ha are not financially viable for the California forest carbon offset program, 80% of the privately owned land (parcels less than 100 ha) in North Carolina is implicitly excluded from participation in these programs by all but the landowners most committed to mitigating climate change, and those landowners were likely to preserve their forests regardless. Montana is different, but still 25% of the privately owned land is distributed in parcels of less than 100 ha and more than 99% in parcels less than 1000 ha. This is hardly the additionality that offset programs seek, i.e. it does not motivate additional carbon sequestration on lands that would not do so in the absence of the offset program. Figure 3 shows the proportion of land and landowners in North Carolina for different parcel sizes, demonstrating the dominance of the state by small holdings. Note that the histograms do include land not currently in forest, which significantly increases the proportion of landowners of small parcel sizes. Over 60% of North Carolina is forested [20] and our interest is in both preserving and increasing forests.

In contrast to the roughly 50% of forest under private ownership in the U.S., over 75% of forest land in Sweden, for example, is privately owned, whereas 0% is privately owned in Russia [21]. While there is clearly great variation among countries, data from the U.S. and Sweden illustrate the importance of the small landowner. According to Haugen et al. [22], the mean size of privately owned productive forest land in Sweden (NIPF—non-industrial private owners) is less than 65 ha, accounting for 50% of the area of productive forest land in Sweden.

Looking at the pattern of land ownership in North Carolina and Sweden illustrates the challenge. Plus, parcelization will likely serve to increase the importance of small land-owners over time [23]. Federal lands comprise 28% of U.S. land [24]. Globally, Obersteiner et al. [25] write about the 1.6 billion people who economically depend on forests. Co-benefits and issues beyond carbon storage will continue to play a major role in land management decisions.

Given sequestration opportunities for terrestrial carbon storage in small holdings, how do we motivate carbon storage given the broad range of land ownership and land management? Encouraging participation requires a detailed understanding of what motivates small landowners, in different regions and different countries; and finding ways to balance our rigorous accounting ideals with the reality of the needed incentives. Gren and Aklilu [26] note pointedly that for forest carbon programs “specific design problems are associated with the heterogeneity of landowners, uncertainty, additionality, and permanence in carbon projects.” The limits to carbon sequestration are not all in the biophysics. Gren and Aklilu suggest that one alternative is to “accept the magnitude of non-additionality and non-permanence and design policy instruments accounting for the deficiencies”. The key for confronting climate change is to motivate establishment of forest where forest does not exist and to motivate the preservation of forest land as forest land.

### Barriers to participation

A number of analyses have looked at potential barriers to participation in forest offset programs (e.g. [13, 26]). Some few have even polled landowners to determine their positions first hand [6, 27]. While many of these studies report most of the same barriers, there is variation by region. Here we discuss briefly some of the prevailing thoughts. Our objective is to understand what then is required of an accounting system that would motivate negative carbon emissions through sequestration in the terrestrial biosphere, especially for a system that considers the interests of small landowners? And, do we need to show these negative emissions on the same balance sheet with positive emissions?

All approaches to forest carbon offsets encounter the problem of adverse selection, i.e. the prospect of conferring offsets to a project that would have taken place regardless of the incentives offered. Projects that are not truly additional discredit the integrity of the program and permit excess emissions elsewhere if they do not generate true additional negative emissions. In a system of practice-based payments it is often that governments are paying for the carbon offsets (although there are some projects with private sponsors) and it is important to be able to show that offset projects actually reduce net carbon emissions.

From 1994 data for the United States, almost 30% of forest landowners were retired and these older landowners owned over 32% of the privately owned land [16]. This means that commitments over 20 years likely involve committing the land beyond the ownership of the current owner. While this may help transition commitments over time for the same property and ensure stability of the project, many landowners are reluctant to commit to agreements for long periods of time. The California forest offset program, for example, has a minimum real commitment of 120–100 years past when the last credit is earned. An agreement that.

Further, the administrative costs of initiating a project are not insignificant and entail many of the same costs, regardless of the size of the project. Initial inventories of the land are scaled, but filing papers and organizing reporting are similar. The earning of credits is often delayed from the costs of listing the project. While it hasn’t been mentioned prominently in the literature, due to many of the programs being quite new, many of the credits earned in forest offset projects are heavily front loaded. That is, the number of earned credits goes down over time, for example when a reforested area begins to mature. With the land committed to a program, little income would be coming into pay for the ongoing protection, inventories, and administrative costs. The value of the land with ongoing costs, little income, and limitations on usage is likely diminished. With ownership turnover typically occurring every few decades, it is not clear what the implications on values and future participation might look like. To build a carbon market, carbon needs to show desirable qualities as a stable object for trading (Liu 2017).

A common concern, additionality, is an essential criterion for credits in all accounting standards and schemes. Additionality is, however, a complex concept. It is essentially a question of causation. Can one relate the emission reduction to a particular incentive? There is, however, a direct impact on environmental integrity from non-additional credits.

Further barriers to entry include the initial process of determining a baseline and reversal risk—never a simple undertaking, especially for small land owners without an analyst or accountant to expedite the process. Muddling through the analyses necessary to evaluate and quantify the baseline and reversal risk outlined in the California protocol, for example, is no easy task. The necessary accounting may be enough to turn many small landowners away.

Even within individual protocols accounting rules can include inconsistencies in the evaluation and award of an offset ton. In the California cap and trade program, for example, land-based carbon uptake is sometimes discounted depending on the value of the land for alternate purposes, i.e. the opportunity cost of choosing to sequester carbon. In essence place matters because of the differences in the value of other goals in land use.

In pay-for-performance systems, issues of baselines, additionality, risk, measurement uncertainty, permanence, verification, and leakage often get treated differently in different protocols and create challenges for fungibility of credits among offset systems (e.g. [28]). A ton is not necessarily a ton [29]. The value of emissions offsets can vary with their duration, their uncertainty, their risk, the buyer’s (renter’s) discount rate, and with the buyer’s (renter’s) expectation of the growth rate of damages from carbon emissions.

One of the issues with current programs is a focus on preserving standing forests and an aversion to harvesting. While maintaining a healthy, standing forest is important, harvesting wood and putting that carbon into long lasting products can sometimes increase the chance that the carbon will be stored for longer than if it remained in the forest, and thus increase the total amount of carbon stored in the biosphere. The half-life of forest products in construction can be up to 100 years and the chance of reversal may be small compared to the risk of insect infestation or fire in a forest. In the California forest offset system landfill carbon is treated inconsistently and the landfill is under-realized as a viable storage place for carbon. Storing carbon in landfills may be less desirable than frugal use of resources and extending the useful lifetime of a product, but the effectiveness of a landfill in carbon storage is still important [30].

Landowners have many different motivations for keeping their forested land forested. Many do not actually want to harvest at all and are more interested in the inherent beauty of the landscape and the ecosystem living in the forest. While it is challenging to place a value on these intangible assets, some studies have shown significant willingness to pay for such environments [31].

Jurisdictions have, by and large, innovated towards more standardization and streamlining of the concept of additionality. Given the fragmentation of the carbon market, many jurisdictions have set about creating their own offset scheme, more appropriate to their circumstances and motivations. Increased ambition in reducing net carbon emissions will likely raise the demand for international trading of offsets, but will also increase scrutiny on the credibility of the additionality determination of offsets [32, p. 23]. This implies a potentially bigger role for international transfers of carbon allowances and credits. As domestic carbon initiatives interact with national commitments, carbon credits with different additionality protocols or demands may significantly hinder linking [32, p. 24].

## Discussion

### The 2050 EZ

In a 2012 paper Richards and Huebner raised the question of the feasibility of designing a forest carbon-offset protocol that provides both reasonable credibility and low transaction costs. They wrote that “It is critical that estimates of offset projects emissions reductions and removals reflect actual contributions” [33, p. 393]. There are serious limitations to a project-based carbon offset strategy [33]. But, is it necessary that debits and credits be symmetric and immediate? And, while we recognize that time matters, that a ton of carbon emitted now is not the opposite of a ton sequestered 10 years from now, there is not an agreed approach for the temporal discounting of emissions and sequestration.

What then are the issues in equating a ton of carbon, in offsetting emissions, in trading of emissions, in evaluating the multiple goals of land management, in defining and measuring negative carbon emissions?

In the case of forest carbon sequestrations, a less stringent accounting approach may increase participation and thus increase ultimate carbon storage, along with achieving greater buy-in for climate friendly policies and the co-benefits that generally accompany forest management for carbon. Can we define “good enough” (Richards and Huebner) while minimizing transaction costs? Olsson et al. [34] have suggested a system wherein some uncertainties are accepted in order to achieve clean development and the participation of countries that are in an early stage of development.

To encourage project participation, protocols must recognize that business-as-usual is market-based, dynamic, and difficult to demonstrate for additionality; and that current decisions affect current actions but cannot guarantee permanence. We need to recognize that place matters because of opportunity costs, risks, and non-carbon, contextual factors. We need straight-forward and non-punitive approaches for dealing with risks and reversals. We also need a comprehensive and consistent treatment of durable wood products and carbon in landfills. In sum, we need clear incentives and simple accounting approaches that encourage participation by diverse participants, including small land holders. We need to confront measuring, reporting and verification at some level.

The simplified 1040EZ filing system for U.S. income taxes may provide insight for a protocol model that balances reward, effort, and risk, while still achieving the overall objectives of standardized protocols and credible results. The 1040EZ tax form in the U.S. is designed for people who have very simple finances or for people who do not want to spend the time and effort for filing their tax returns personally. People who use this form may end up paying a bit more in taxes, but are saved the task of compiling the data needed to document deductions, expenses, and other related tax transactions. Its appeal is its simplicity.

We propose the idea of a “2050 EZ protocol” for forest carbon sequestration—as a complement to existing protocols. We propose developing a very simple process for enrolling land in a forest offset program that lowers the barriers that discourage small landowners from participating. In the next section we outline the main tenets of such a program and suggest strategies for reducing administrative overhead, lowering transaction costs, and reducing commitment lengths; but preserving environmental integrity, albeit with some accepted analytical uncertainty. The earned carbon credits could be less than for the currently available protocols but the lower point of entry might increase participation and provide a vital contribution to the overall goal of mitigating climate change. Our discussion alludes to the multiple barriers to participation but focuses on the need for long-term or “permanent” commitments. We note that forest offset programs do not require “permanence” to be effective. Marland et al. [35] described a system of carbon rentals and Lintunen and Rautiainen [36] analyze the equivalency of a rental approach with a traditional “subsidize-and-tax model”.

### Goals

The challenge then, is to develop a simple protocol that many small landowners could implement for renewable short-term contracts with minimum transaction costs. A protocol that would encourage and reward retention of forests and storing carbon in forests. We will call this process the 2050EZ process in recognition of the US tax form and the target date of many emissions scenarios. There are at least two reasons that the 1040EZ tax form is worthwhile to the U.S. Internal Revenue Service. First, it is everyone’s responsibility to pay their taxes so that everyone pays their “fair share”, and second, the government needs the money to function. While the percentage of people filing the 1040EZ paper form is only a small fraction of total filings (about 13% of total paper returns), that still accounts for over 5.5 million people in 2010 [37]. The impact on the government budget is important. We look at the 2050 EZ form in the same way. Everyone has a responsibility to contribute to solving the problem of human-caused climate change (we all benefit regardless where the fault may lie), and if enough people participate it can make a big impact on the sequestration of carbon from the atmosphere. Participation of small landowners would also provide a solid base of climate policy supporters who would feel vested in the solution and feel tangible benefits from their investiture. We begin with the two basic tenets of the 2050 EZ protocol:

1. The protocol should enable widespread participation in forest carbon offsets without high transaction costs that disproportionately disadvantage small landowners.
2. The protocol should produce fewer credits per project than the standard protocols, to account for the increased uncertainty of projects that do not fit current definitions of additionality and permanence, or that have higher measurement uncertainty.

The goal is to create more buy-in from landowners with small land holdings. The projects need to be effective, but the more buy-in the better for long-term and wide-spread support of climate friendly and sustainable policies (see, e.g. [38]). Landowners who hold large areas of land or particularly productive lands should be encouraged to participate in the more rigorous accounting of the full protocols. Those protocols have more strict requirements and the projects under those guidelines may be more likely to represent permanent reductions that have more accurate accounts of the carbon sequestered from the atmosphere. The 2050 EZ would likely produce fewer dollars per hectare over the life of a project, but there are so many parameters and uncertainties in the calculations that it is challenging to ensure that. With these fundamental objectives in mind and the basic approaches outlined, we turn discussion to several topics that warrant more discussion. In the past, short-term storage has caused some debate. We offer two arguments to support the use of short-term commitments and the value of short term storage. As a simple model, we can look at the effect of short term storage in an intuitive way. More realistic models would follow the same basic trends. Suppose we assume that a single product might be produced at a constant rate (J) and decay exponentially (first order decay—unrealistic, but a beginning). Then the stock of that product would follow the following model.

1 $$\frac{{d \left[ {Stock} \right]}}{dt} = J - r \cdot [Stock]$$

The rate of decay is *r* and the half-life ($T_{hl}$) of the product is $T_{hl} = \frac{\ln \left( 2 \right)}{r}$. If we replace *r* in Eq. (1), we get

2 $$\frac{{d \left[ {Stock} \right]}}{dt} = J - \frac{\ln \left( 2 \right)}{{T_{hl} }} \left[ {Stock} \right]$$

In steady state, the total stock reaches a value of

3 $$\left[ {Stock} \right]_{Steady State} = \frac{{J T_{hl} }}{\ln \left( 2 \right)}$$

Equation 3 shows that the steady state stock of carbon in the product is directly proportional to the half-life of the product. Now suppose that we extend the half-life of the product by 10% while the rate of production is unchanged. This translates into a 10% increase in the total, steady-state stock of carbon contained in that product. So even though the carbon stored in a single unit of product is temporary, the increase in half-life results in more carbon retained out of the atmosphere. This is where the value of short-term storage and the value of short-term projects can make a difference. We need to avoid thinking of each forest carbon project in isolation, but instead consider its contribution to the larger program comprised of multiple projects.

If we look more carefully at the accumulation of carbon through short-term projects, we can look at forests themselves as examples. Forests are valued as vital stocks of carbon. Forests can sequester large quantities of carbon, and with careful management, can store even more. In fact, a simple calculation similar to the one above shows that if the average half-life of harvested wood from a forest exceeds the rotation time of the harvest, there will be more carbon from the forest in its products than in the forest itself.

**Fig. 4:**
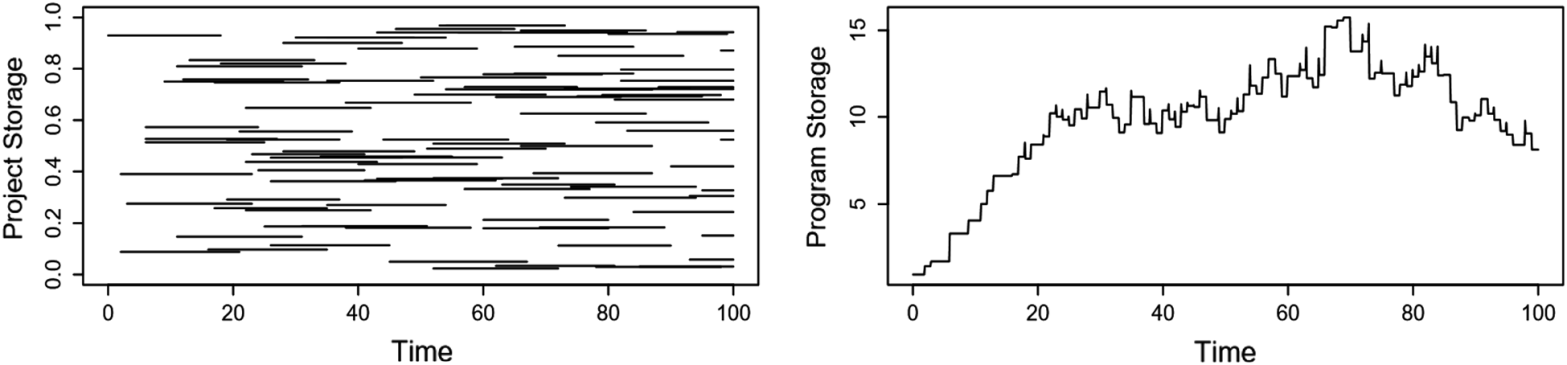
The left panel simulates a series of hypothetical, short term, small projects that begin and end over intervals of 10 to 30 years. The projects are scaled to hold between 0 and 1 unit of carbon. The right panel shows the accumulation of carbon for all of the projects added together

Each tree in a forest has a finite lifetime, seen as short-term carbon storage. But, trees do not live forever. A forest is a collection of short-term projects that we consider to be a long-term storage of carbon. In the same way, short-term carbon sequestration project can be considered as a part of a larger long-term program. Figure 4 shows a simulation of a conglomeration of short term projects of varying length and magnitude and how their combination comprises a relatively steady, long term program.

In the “Discussion” section above we characterized the nature of land ownership in both the United States and Sweden, taking note of the distribution of parcel sizes and the fact that small parcels collectively comprise a large fraction of the total area of forested land. If the barriers to entry into a forest carbon offset program are too great for all of these small landowners to reasonably consider joining such a forest offset program, we should consider accounting protocols that lower the barrier for entry.

Two means to potentially increase the participation of small land owners include shorter contracts of “permanence” for only, for example, 20 years, and a simple standard measure of forest carbon stocks. Shortening the contract length to 20 years at first glance seems like a risk, as a land owner could much more quickly and easily back out of the program. However, the 100-year time commitment is likely more of a gate- keeping obstacle that prevents land owners from joining offset protocols to begin with. The generations-long commitment is unlikely to appeal to those not part of a conservancy or other organization that will outlast them. It could be more beneficial to offset programs to gain the interest and participation of many land owners with shorter contracts, and then to provide enough incentives for them during the timeline of the contract that they consider extension for another term afterward.

Second, the use of a simple approximation of carbon based on data such as LiDAR or other canopy-cover measures that are fast, simple, and inexpensive for a given small plot of land could significantly decrease the upfront and monitoring costs to the landowner for plotting and measuring. While this could increase small landowner participation, this approximation must be conservative enough that it does not appeal to land owners with large enough tracts of land. For larger projects, lower uncertainty is desirable and they can bear the cost of more rigorous accounting. To create the model for the 2050 EZ, we begin with several ideas that could drive the development of the model.

1. We assume a single forest project type—a forest. The three categories in existing protocols—reforestation, improved forest management, and avoided conversion—might be considered different phases of the same forest. First the forest must begin, then it must grow, and finally we try to avoid having it revert back to an un-forested state. As a consequence, offsets need to provide incentives for growing a forest, growing it faster, and for keeping it as forest.
2. We credit carbon stocks rather than carbon relative to a baseline. Baselines are both uncertain in long term forecasting and are generally based on common practice. In order to gain participation from more people, credits in the EZ form could be given for the total tonnage of carbon held in the forest and forest products and thus kept out of the atmosphere. The incentive for improving the management of the forest is simply a consequence that it sequesters additional tonnes of carbon and is therefore awarded more credits. Increases in stocks could be rewarded more than retention of stocks.
3. We propose short term contracts. The shorter time reduces the permanence requirement of each individual project but increases the appeal for more landowners. If a significant fraction of the projects re-enroll at the end of their term or if there is a constant influx of new projects, the total stock of carbon currently involved in the program at any one time would be significant. In the same way that a single tree in a permanent forest is transient, the program as a whole could be considered permanent even though individual projects may enter and leave.

For each enrolled project, the total carbon inventory of the forest needs to be estimated initially and at intervals to determine the credits earned. In between measurements, models could be used to estimate intermediate values that are then reconciled at the next inventory measurement.

The value of a ton of sequestered carbon could be estimated based on tonne-years. There would be incentives for each year that a ton of carbon was retained out of the atmosphere. Long-lived wood products would be more attractive than short-lived products. Risks and uncertainties would be unnecessary to consider since credits would be only given for credits already earned. Penalties are only applied for early termination of a contract.

## Looking forward

Our intent in this paper is not to offer a fully developed protocol for carbon sequestration credits but to lay the ground work and stimulate discussion of ways to increase the incentives for participation and thus to increase the total amount of carbon sequestered in the biosphere. The urgency of addressing climate change suggests a need to increase and protect the mass of sequestered carbon. The final question in implementing a 2050 EZ form for carbon sequestration activities would be to detail the best approach for specific implementation. Selling carbon credits on the open market works for large holdings. But buying and selling credits from specific projects is cumbersome; and we must consider how the value of credits from different projects are equated. The Family Forest Carbon Program of the American Forest Foundation and the Nature Conservancy is similarly pursuing to remove the barriers that family forest owners encounter in many forest carbon protocols [39].

The 2050 EZ credits should form a pool of credits that have a defined equivalence to each other and are compensated equally. One method of monetizing the credits is through tax credits. Another is to treat them as a scheduling process. The first credits that go in would be the first paid out. Since the credits are determined each year, projects would be assured of being paid for their year x credits before any year x + 1 credits are paid.

Clearly there are multiple possibilities for specific implementation of easy accounting but likely fewer credits. This paper offers a framework for a new approach and does not presume to construct an entire protocol Policy is created through discourse and incremental development. We offer a first step.

The end goal is to reduce atmospheric CO_2_ levels by reducing the rate of emissions and increasing the rate of sequestration. In an ideal world, all landowners and stake holders would be in support of this goal. In reality, we need to motivate and incentivize participation. We need to develop programs that have appeal and gain buy-in from many different people with varying motivations. That means that not only should they want to participate, they should want to continue participating. To appeal to a broad set of land-owners, it is not clear to us that a single one-size-fits-all program will be as effective as creating multiple programs to appeal to specific audiences.

In this scenario, we are not trying to tie up land by buying people out. Some people want to tie their land up in programs that prevent development and create a lasting effect long after they pass on. The system of conservation easements and conversion to public lands is a great option for those people. Others need an alternative and respond negatively to the idea that someone is effectively buying them out of their choices of what can happen on their land. Instead of buying them out, we propose to get them to buy in, repeatedly.

## Conclusion

Here we have laid some ground work and proposed developing a system that relies on short-term agreements and easy accessibility for encouraging the accumulation and retention of carbon in forests. This system will likely not be as effective in generating carbon credits per unit of land area in each project as a more rigorous program, but we propose getting people to buy in and become part of an ongoing system. If they do not want to keep participating, then we have created an insufficiently motivating system. We aim on obtaining lots of participation on small efforts. The challenge is balancing program stringency and participation.

We propose that shorter contracts will be more attractive to small land owners but shorter contracts also offer flexibility for the program administration. The terms of the agreements could be modified as new ideas emerge or as the climate changes. The ideal agreements now may not be ideal 50 years in the future and we may not want to bind ourselves to a system that may not mesh with reality at that time.

Finally, we note that one of the ways to move toward more effective climate-stabilizing policies is to get more people vested in short-term outcomes. While a program aimed at small landowners may not sequester as much carbon per landowner as existing programs, each of those landowners gets the same number of votes in elections. Participation in a small program now may turn into support for broader programs later.

## Data Availability

The raw data used in the work are all clearly referenced and available online. The data used to produce the figures is available upon request.

## Abbreviations

CO_2_: carbon dioxide
LiDAR: light detection and ranging

## References

[1] IPCC. Special report on global warming of 1.5 °C, Intergovernmental Panel on Climate Change. 2018. https://www.ipcc.ch/SR15/. Accessed 20 Nov 2019

[2] United Nations. Paris Agreement. 2015. https://unfccc.int/sites/default/files/english_paris_agreement.pdf. Accessed 20 Nov 2019

[3] Gasser T, Guivarch C, Tachiiri K, Jones CD, Ciais P (2015). Negative emissions physically needed to keep global warming below 2 °C. Nat Commun.

[4] Peters GP, Andrew RM, Boden T, Canadell JG, Ciais P, Corinne Le Quéré G, Marland MR Raupach, Wilson C (2012). The challenge to keep global warming below 2 °C. Nat Clim Change.

[5] Obersteiner M, Bednar J, Wagner F, Gasser T, Ciais P, Forsell N, Frank S, Havlik P, Valin H, Janssens IA, Peñuelas J, Schmidt-Taub G (2018). How to spend a dwindling greenhouse gas budget. Nat Clim Change.

[6] Butler BJ, Hewes JH, Dickinson BJ, Andrejczyk K, Butler SM, Markowski-Lindsay M (2016). Family forest ownerships of United States, 2013: findings from the USA Forest Service’s National Woodland Owner Survey. J Forestry.

[7] Galik CS, Mobley ML, Richter DdeB (2009). A virtual “field test” of forest management carbon offset protocols: the influence of accounting. Mitig Adapt Strateg Glob Change.

[8] FAO. Global Forest Resources Assessment 2015, Food and Agriculture Organization of the United Nations. 2015. http://www.fao.org/3/a-i4808e.pdf. Accessed 13 Mar 2018.

[9] Bayon R, Hawn A, Hamilton K (2013). Voluntary carbon markets: an international business guide to what they are and how they work.

[10] Mercker D. The business of carbon credit trading for forest landowners. The University of Tennessee Institute of Agriculture report 09-0191 W217-4/09. 2009. https://extension.tennessee.edu/publications/Documents/W217.pdf. Accessed 20 Nov 2019

[11] Zeng N (2008). Carbon sequestration via wood burial. Carbon Balance Manag.

[12] California. The California Global Warming Solutions Act, State of California Assembly Bill 32. 2006. https://www.arb.ca.gov/cc/ab32/ab32.htm. Accessed 20 Nov 2019

[13] Kerchner CD, Keeton WS (2015). California’s regulatory forest carbon market: viability for northeast landowner. For Policy Econ.

[14] Ruseva T, Marland E, Szymanski C, Hoyle J, Marland G, Kowalczyk T (2017). Additionality and permanence standards in California’s Forest Offset Protocol: a review of project and program level implications. J Environ Manag.

[15] Osborne T, Shapiro-Garza E (2017). Embedding carbon markets: complicating commodification of ecosystem services in Mexico’s forests. Ann Am Assoc Geogr.

[16] Birch TW (1996). Private forest-land owners of the United States, RB-NE-134..

[17] NC ONE Map. http://www.nconemap.com. Accessed 1 Apr 2018.

[18] Montana. Geographic information clearinghouse, Montana State Library. 2018. http://geoinfo.msl.mt.gov. Accessed 20 June 2018.

[19] CGIA. The North carolina center for geographic information and analysis, NC ONE MAP. 2018. http://www.nconemap.com. Accessed Apr 2018

[20] Brown M, Lambert S (2016). Forests of North Carolina, 2014. Resource Update FS-101.

[21] FAO. FRA 2010 country reports. 2010. http://www.fao.org/forestry/fra/67090/en. Accessed 20 Nov 2019

[22] Haugen K, Karlsson S, Westin K (2016). New forest owners: change and continuity in the characteristics of Swedish Non-industrial Private Forest Owners (NIPF Owners) 1990–2010. Small-scale Forestry.

[23] Hatcher JE, Straka TJ, Greene JL (2013). The size of forest holding/parcelization problem in forestry: a literature review. Resources.

[24] CRS. Federal land ownership: overview and data. Document R42346. 2017. https://fas.org/sgp/crs/misc/R42346.pdf. Accessed 9 July 2018.

[25] Obersteiner M, Bednar J, Wagner F, Gasser T, Ciais P, Forsell N, Frank S, Havlik P, Valin H, Janssens IA, Peñuelas J, Schmid-Traub G (2018). How to spend a dwindling greenhouse gas budget. Nat Clim Change.

[26] Gren I-M, Aklilu (2016). Policy design for forest carbon sequestration: a review of the literature. Forest Policy Econ.

[27] Thompson DW, Hansen EN (2012). Factors affecting the attitudes of nonindustrial private forest landowners regarding carbon sequestration and trading. J Forestry.

[28] Riehl B, Wang G, Eshpeter S, Zhang H, Innes JL, Li N, Li J, Niles JO (2016). Lessons learned in mandatory carbon market development. Int Rev Environ Resour Econ.

[29] Lee CM, Lazarus M, Smith GR, Todd K, Weitz M (2013). A ton is not always a ton: a road-test of landfill, manure, and afforestation/reforestation offset protocols in the U.S. carbon market. Environ Sci Policy.

[30] Micales JA, Skog KE (1997). The decomposition of forest products in landfills. Int Biodeterior Biodegrad.

[31] Nielsen-Pincus M, Sussman P, Bennett DE, Gosnell H, Parker R (2017). The influence of place on the willingness to pay for ecosystem services. Soc Nat Resour.

[32] WorldBank. Carbon credits and additionality: past, present, and future, PMR Technical Note 13. Partnership for Market Readiness, World Bank, Washington D.C. License: Creative Commons Attribution CC BY 3.0 IGO. 2016. http://documents.worldbank.org/curated/en/407021467995626915/pdf/105804-NWP-PUBLIC-PUB-DATE-5-19-2016-ADD-SERIES.pdf. Accessed 20 Nov 2019

[33] Richards K, Huebner GE (2012). Evaluating protocols and standards for forest carbon-offset programs, part A: additionality, baselines and permanence. Carbon Manag.

[34] Olsson A, Grönkvist S, Lind M, Yan J (2016). The elephant in the room - A comparative study of uncertainties in carbon offsets. Environ Sci Policy.

[35] Marland G, Fruit K, Sedjo R (2001). Accounting for sequestered carbon: the question of permanence. Environ Sci Policy.

[36] Tahvonen O, Rautiainen A (2016). Economics of forest carbon storage and the additionality principle. Resour Energy Econ.

[37] Collins B. Projections of Federal tax return filings: calendar years 2011–2018. 2019. https://www.irs.gov/pub/irs-soi/12rswinbulreturnfilings.pdf. Accessed 04 Sept 2019.

[38] Cowie A, Eckard R, Eady S (2012). Greenhouse gas accounting for inventory, emissions trading, and life cycle assessment in the land-based sector: a review. Crop Pasture Sci.

[39] American Forest Carbon Program. 2019. https://www.forestfoundation.org/family-forest-carbon-program.

